# The Africans in America study demonstrates that subclinical cardiovascular risk differs by etiology of abnormal glucose tolerance

**DOI:** 10.1038/s41598-022-19917-8

**Published:** 2022-10-10

**Authors:** Annemarie Wentzel, M. Grace Duhuze Karera, Arielle C. Patterson, Zoe C. Waldman, Blayne R. Schenk, Lilian S. Mabundo, Christopher W. DuBose, Margrethe F. Horlyck-Romanovsky, Anne E. Sumner

**Affiliations:** 1 Section on Ethnicity and Health, Diabetes, Endocrinology, and Obesity Branch, National Institute of Diabetes, Digestive and Kidney Diseases, National Institutes of Health, Bethesda, MD USA; 2 Hypertension in Africa Research Team (HART), North-West University (NWU), Potchefstroom, North-West South Africa; 3 South African Medical Research Council, Unit for Hypertension and Cardiovascular Disease, North-West University, Potchefstroom, North-West South Africa; 4 National Institute of Minority Health and Health Disparities, National Institutes of Health, Bethesda, MD USA; 5 Institute of Global Health Equity Research, University of Global Health Equity, Kigali, Rwanda; 6 Department of Health and Nutrition Sciences, Brooklyn College, City University of New York, New York, USA

**Keywords:** Physiology, Biomarkers, Cardiology, Endocrinology, Risk factors

## Abstract

Abnormal-glucose tolerance (Abnl-GT) is due to an imbalance between β-cell function and insulin resistance (IR) and is a major risk factor in cardiovascular disease (CVD). In sub-Saharan Africa, β-cell failure is emerging as an important cause of Abnl-GT (Abnl-GT-β-cell-failure). Visceral adipose tissue (VAT) volume and hyperlipidemia are major contributors to CVD risk when Abnl-GT is due to IR (Abnl-GT-IR). Yet, the CVD profile associated with Abnl-GT-β-cell failure is unknown. Therefore, our goals in 450 African-born Blacks (Male: 65%; Age: 39 ± 10 years; BMI 28 ± 5 kg/m^2^), living in America were to: (1) determine Abnl-GT prevalence and etiology; (2) assess by Abnl-GT etiology, associations between four understudied subclinical CVD risk factors in Africans: (a) subclinical myocardial damage (high-sensitivity troponin T (hs-cTnT)); (b) neurohormonal regulation (N-terminal pro-Brain-natriuretic peptide (NT-proBNP)); (c) coagulability (fibrinogen); (d) inflammation (high-sensitivity C-reactive protein (hsCRP)), as well as HbA_1c_, Cholesterol/HDL ratio and VAT. Glucose tolerance status was determined by the OGTT. IR was defined by the threshold at the lowest quartile for the Matsuda Index (≤ 2.97). Abnl-GT-IR required both Abnl-GT and IR. Abnl-GT-β-cell-failure was defined as Abnl-GT without IR. VAT was assessed by CT-scan. For both the Abnl-GT-β-cell-failure and Abnl-GT-IR groups, four multiple regression models were performed for hs-cTnT; NT-proBNP; fibrinogen and hsCRP, as dependent variables, with the remaining three biomarkers and HbA_1c_, Cholesterol/HDL and VAT as independent variables. Abnl-GT occurred in 38% (170/450). In the Abnl-GT group, β-cell failure occurred in 58% (98/170) and IR in 42% (72/170). VAT and Cholesterol/HDL were significantly lower in Abnl-GT-β-cell-failure group vs the Abnl-GT-IR group (both *P* < 0.001). In the Abnl-GT-β-cell-failure group: significant associations existed between hscTnT, fibrinogen, hs-CRP, and HbA_1c_ (all *P* < 0.05), and none with Cholesterol/HDL or VAT. In Abnl-GT-IR: hs-cTnT, fibrinogen and hsCRP significantly associated with Cholesterol/HDL (all *P* < 0.05) and NT-proBNP inversely related to fibrinogen, hsCRP, HbA_1c_, Cholesterol/HDL, and VAT (all *P* < 0.05). The subclinical CVD risk profile differed between Abnl-GT-β-cell failure and Abnl-GT-IR. In Abnl-GT-β-cell failure subclinical CVD risk involved subclinical-myocardial damage, hypercoagulability and increased inflammation, but not hyperlipidemia or visceral adiposity. For Abnl-GT-IR, subclinical CVD risk related to subclinical myocardial damage, neurohormonal dysregulation, inflammation associated with hyperlipidemia and visceral adiposity.

**ClinicalTrials.**gov Identifier: NCT00001853.

## Introduction

Abnormal glucose tolerance (Abnl-GT) occurs due to an imbalance between β-cell function and insulin resistance (IR) and is an independent risk factor for cardiovascular disease (CVD)^1^. Whether the CVD risk profile associated with Abnl-GT is dependent on etiology, is unknown. In Abnl-GT-IR, hyperlipidemia, visceral adiposity and low-grade inflammation contribute to CVD development^2^. Yet, the degree of CVD risk and the determinants of CVD risk in Abnl-GT-β-cell failure remains to be determined. As African-born Blacks appear to have a high prevalence of Abnl-GT-β-cell failure, working with this population provides an opportunity to explore the subclinical CVD risk profile in Abnl-GT-β-cell failure and potentially gain better insight regarding subclinical CVD manifestation, and possibly diagnostic and therapeutic approaches^3,4^.

To understand the nature of sub-clinical CVD risk profiles in both Abnl-GT-β-cell-failure and Abnl-GT-IR, our investigation moved beyond standard risk profiles and markers, and included (1) subclinical myocardial strain and damage (high sensitivity troponin T (hs-cTnT)), (2) neurohormonal homeostasis (amino-terminal pro-Brain natriuretic peptide (NT-proBNP)), (3) coagulability (fibrinogen), and (4) inflammation (high sensitivity C-reactive protein (hsCRP)).

High-sensitivity cardiac troponin T (hs-cTnT) is a marker of subclinical myocardial damage, strain and ischemia^5^. Higher hs-cTnT is also a predictor of incident diabetes^6^ and associated with chronic hyperglycemia^7^. Subclinical myocardial strain and damage, reflected by elevated hs-cTnT, may occur across the continuum of hyperglycemia^7,8^. Particularly, when hs-cTnT levels exceed 14 ng/mL, it is defined as subclinical myocardial damage^9^. However, whether such increases in subclinical myocardial strain and possible damage occurs, and associates differently with the other markers of subclinical CVD risk, including lipids, adiposity and glycemia, based on Abnl-GT etiology, is unknown.

NT-proBNP shows a significant relationship with hs-cTnT, specifically in African-descent populations^8,10^. NT-proBNP is a marker of neurohormonal homeostasis, counteracting sympathetic nervous system activity (SNS) and the renin–angiotensin–aldosterone system (RAAS) in response to increased volume load and myocardial strain^11^. NT-proBNP is also involved in glucose and lipid metabolism^12^ and lower levels of NT-proBNP predicted worse CVD outcomes in hyperglycemia^13^, possibly indicative of disrupted neurohormonal homeostasis in hyperglycemia^7^.

A marker of coagulation and inflammation that significantly relates to hs-cTnT^14^ and NT-proBNP^15^, is fibrinogen^16,17^. Higher fibrinogen predicted increased CVD risk in diabetes^18^. Additionally, increased hsCRP, the most frequently applied inflammatory marker, indicated increased CVD risk, and has been implicated in both subclinical myocardial damage and diabetes^7,19,20^.

Therefore, our goals in African-born Blacks living in America were to: describe and compare the subclinical CVD risk profiles in Abnl-GT-β-cell failure vs Abnl-GT-IR, by: (a) determining the prevalence and etiology of Abnl-GT; (b) assessing the associations between: (1) hs-cTnT (subclinical myocardial strain and damage) (2) NT-proBNP (neurohormonal dysregulation); (3) fibrinogen (increased coagulability); (4) hsCRP (increased inflammation), and the association of glycated hemoglobin A1c (HbA_1c_), Cholesterol/HDL ratio and visceral adiposity with these four subclinical CVD risk markers.

## Methods

### Population

The Africans in America study is a cross-sectional, clinical protocol designed to assess both the cardiometabolic and social determinants of health of African-born Blacks living in the United States^21–24^. Recruitment was achieved by advertisements in newspapers, flyers, social media, and posters at community events. The protocol was approved by the Institutional Review Board of the National Institutes of Health, National Institute of Diabetes, Digestive and Kidney Diseases (NIDDK) (ClinicalTrials.gov Identifier: NCT00001853). Prior to participation all enrollees provided written informed consent. All methods were performed according to the relevant ethical, clinical and scientific research guidelines and regulations.

To determine eligibility for a screening visit at the NIH Clinical Center a telephone interview was conducted. During the telephone interview, the prospective enrollee had to self-identify as healthy, deny a history of diabetes and confirm that both they and their parents self-identified as Black and were born in a sub-Saharan African country.

Five-hundred and five African-born Blacks currently living in metropolitan Washington, DC completed the telephone interview and proceeded to the screening visit for a history, physical, electrocardiogram and routine blood tests. Questionnaires determined family history of diabetes, use of blood pressure medication and current smoking status.

Twenty-eight individuals did not proceed from the Screening Visit to the OGTT. Reasons for exclusion were anemia (n = 8), elevated liver transaminases (n = 1), hypothyroidism (n = 1), pregnancy (n = 3), IV access issues (n = 4), declined blood draw (n = 2) and scheduling conflicts (n = 9)**.**

Following OGTT analysis, 27 (n = 27) individuals were excluded. One participant had a fasting insulin concentration of 172 pmol/L (normal < 30 pmol/L) and was diagnosed with extreme insulin resistance. Eight other individuals had either a missing value for glucose or insulin during the multi-sampled OGTT, and therefore the Matsuda Index could not be calculated.

Four participants had hs-cTnT, and two participants had NT-proBNP below detectable limits, 1 participant did not have a fibrinogen value and 11 participants did not have a visceral adipose tissue scan and were therefore excluded from all further analyses.

**Figure 1:**
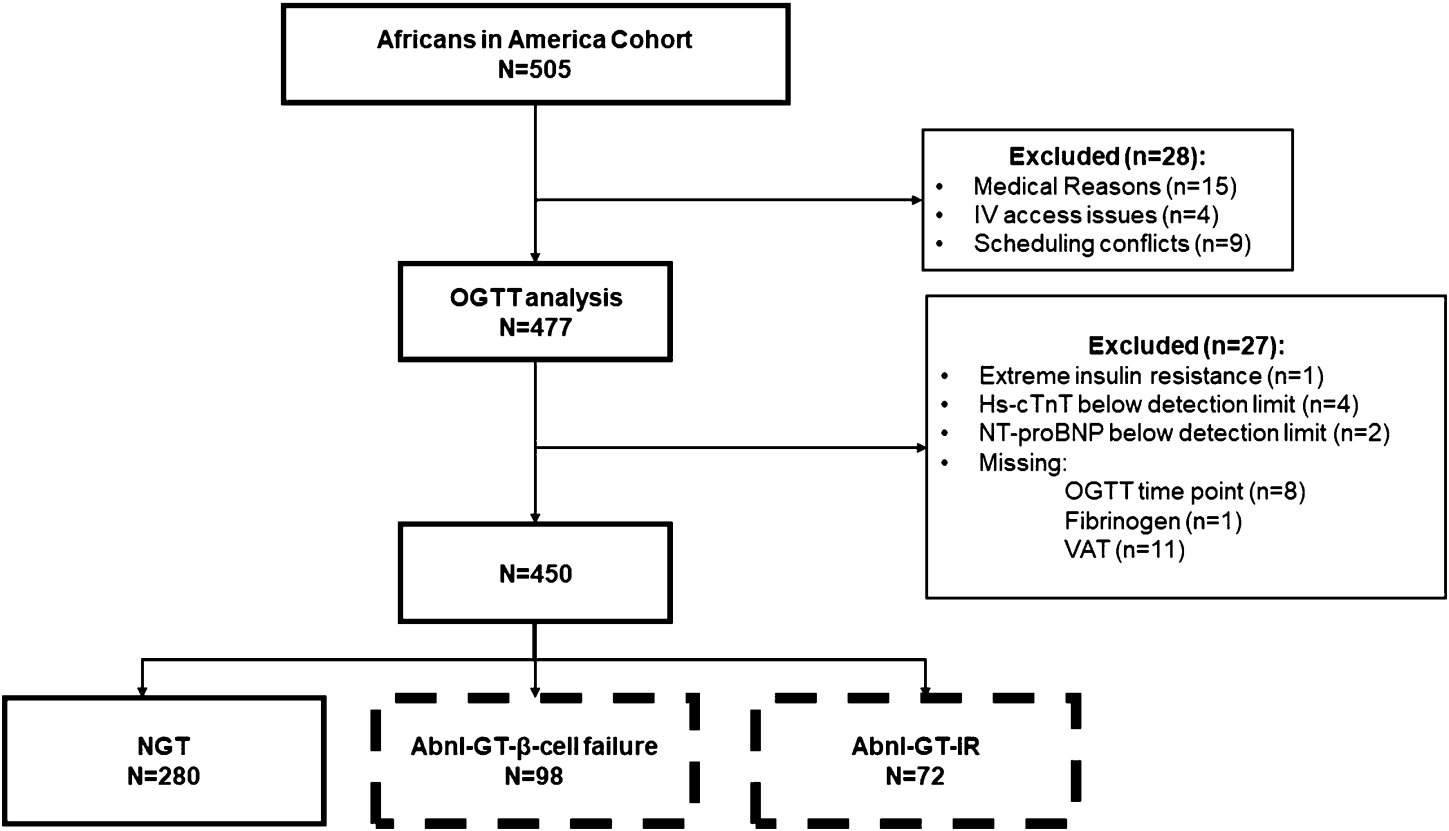
Flow diagram for recruitment and determination of glucose tolerance groups.

The remaining 450 participants were studied (Fig. 1).

### Oral glucose tolerance test visit

After a 12 h overnight fast, participants arrived at the NIH Clinical Center at 7AM. Weight, height, waist circumference (WC) and blood pressure (BP) were measured. Hypertension (HT) was defined as SBP ≥ 130 mmHg and/or DBP ≥ 80 mmHg^25^. Obesity was defined as BMI ≥ 30 kg/m^2^^26^. WC was measured at the superior border of the iliac crest at the end of expiration^27^, and the mean of three values was recorded.

Baseline blood samples were obtained for fasting glucose, insulin, glycated hemoglobin A_1c_ (HbA_1c_), high-sensitivity cardiac troponin T (hs-cTnT), amino-terminal pro-brain natriuretic peptide (NT-proBNP), fibrinogen, high-sensitivity C-reactive protein (hsCRP), cholesterol, triglycerides and HDL cholesterol. Estimated glomerular filtration rate (eGFR) was calculated according to the chronic kidney disease epidemiology (eGFR-CKD-EPI) collaboration formula^28^. Post-Glucola consumption (Trutol 75, Custom Laboratories) blood samples were taken at 0.5 h, 1 h, 2 h to determine glucose and insulin concentrations, which were used to calculate the Matsuda Index.

After the OGTT, a computerized tomographic (CT) scan (Siemens and Somatom Force Scanner) with adipose windows designed to measure visceral adipose tissue (VAT), was performed^29^.

### Glucose tolerance status

A diagnosis of abnormal glucose tolerance (Abnl-GT) required: FPG ≥ 100 mg/dL (5.6 mmol/L) and/or 2 h glucose ≥ 140 mg/dL (7.8 mmol/L). A diagnosis of diabetes required FPG ≥ 126 mg/dL (7.0 mmol/L) and/or 2 h glucose ≥ 200 mg/dL (11.1 mmol/L).

### Insulin resistance status

Insulin resistance status was determined by Matsuda Index^30^:

$$\left(\frac{\mathrm{10,000}}{\sqrt{fasting \,glucose \times fasting\, insulin \times mean \,glucose \times mean\, insulin}}\right).$$

Insulin resistance was defined as the lowest quartile for our population distribution of Matsuda Index (≤ 2.97) and insulin sensitive as Matsuda Index > 2.97.

### Insulin secretion status

Insulin secretion was determined by the insulinogenic index (ISI) = $\frac{AUC\, for\, Insulin \,from\, 0\, to\, 120 \,\mathrm{min}}{AUC\, for \,Glucose\, from\, 0 \,to\, 120\, \mathrm{min}}$^24^.

### Degree of β-cell compensation

The oral disposition index (DI) is a measure of β-cell function adjusted for insulin sensitivity^31^. The DI was calculated as the product of the ISI and Matsuda Index: DI = (ISI) × (Matsuda Index)^31^. The oral DI gives an indication of each individual’s β-cells’ ability to overcome their degree of insulin resistance.

### Assignment by Glucose tolerance and insulin resistance status (Fig. 1)

Participants were categorized first by the absence or presence of Abnl-GT, and then within the Abnl-GT group, by the absence or presence of insulin resistance.

#### Normal glucose tolerant total group (NGT)

Normal glucose tolerance (Matsuda Index > 2.97).

#### Abnormal glucose tolerance due to β-cell-failure (Abnl-GT-β-cell-failure)

Abnl-GT due to β-cell-failure, as Abnl-GT occurred in the absence of insulin resistance (Matsuda Index > 2.97).

#### Abnormal glucose tolerance due to insulin resistance (Abnl-GT-IR)

Abnl-GT due to insulin resistance, as Abnl-GT occurred in the presence of insulin resistance (Matsuda Index ≤ 2.97).

### Biochemical analyses and calculations

Hemoglobin and hematocrit were measured in EDTA-anticoagulated whole blood (Sysmex XE-5000). Insulin was measured in serum and glucose, cholesterol, triglyceride, HDL, fibrinogen and hsCRP in plasma using the Roche Cobas 6000 analyzer (Roche Diagnostics, Indianapolis, IN, USA). LDL was calculated using the Friedewald equation^32^. Cholesterol/HDL ratio was calculated. HbA_1c_ values were determined by HPLC using National Glycohemoglobin Standardization Program (NGSP)-certified instruments manufactured by BioRad Laboratories (Hercules, CA, USA).

Hs-cTnT and NT-proBNP were measured in stored EDTA-plasma samples (− 80 °C) and analyzed in duplicate. hs-cTnT was measured using the human-high-sensitivity-cTnT ELISA sandwich assay from MyBioSource, with a detection range of 0.5–150 ng/L (MyBioSource, CA, USA). Elevated hs-cTnT was defined as the 99th percentile of 14 ng/L, indicating subclinical myocardial damage, in an apparently healthy population, aged 20–70 years^9^. Therefore, in this study hs-cTnT concentrations of ≥ 14 ng/L were considered elevated and indicative of subclinical myocardial damage.

NT-proBNP was measured with the human-NT-proBNP ELISA sandwich assay from Abcam, with a detection range of 21.9–1400 pg/mL (Abcam, Boston, MA, USA). As disrupted neurohormonal homeostasis may be reflected by NT-proBNP levels < 50 pg/mL^7^, NT-proBNP < 50 pg/mL were considered low, and indicative of subclinical neurohormonal dysregulation.

### Statistical analysis

Normal distribution of data was assessed within the total cohort and Abnl-GT-β-cell-failure and Abnl-GT-IR groups separately, by the Kolmogorov–Smirnov test. Data for cardiovascular and inflammatory markers are presented as the median and interquartile ranges, all other data are presented as mean ± SD for continuous variables and percentage for categorical variables. Unpaired t-tests compared continuous variables, and categorical variables were compared by Chi-square test, between Abnl-GT-β-cell failure versus Abnl-GT-IR. One-way ANOVA with Bonferroni corrections for multiple comparisons were used to compare the three African regions of origin. Hs-cTnT, NT-proBNP, fibrinogen and hsCRP were log-transformed, and the natural logarithmic transformations were used in regression analyses.

Partial correlation and sensitivity analyses were done with CVD risk biomarkers and demographic, socio-economic and health behavior variables, including hypertension and diabetes status, and use of antihypertensive medication to determine the most physiologically justified and statistically relevant a priori covariates. A priori confounders included age, sex, hypertension status, and smoking in all models. As the groups were divided according to insulin resistance status, we did not adjust for Matsuda Index in the regression analyses.

To assess the relationship between cardiovascular and inflammatory markers, glycemia, lipids and adiposity in each Abnl-GT group separately, multiple linear regression, adjusted for a priori covariates, in four separate models within Abnl-GT-β-cell failure and Abnl-GT-IR, were performed.

#### Model 1

Dependent variable was hs-cTnT, with independent variables NT-proBNP, fibrinogen, hsCRP, HbA_1c_, Cholesterol/HDL ratio, VAT, eGFR, African region of origin and a priori covariates.

#### Model 2

Dependent variable was NT-proBNP, with independent variables hs-cTnT, fibrinogen, hsCRP, HbA_1c_, Cholesterol/HDL ratio, VAT, eGFR, African region of origin and a priori covariates.

#### Model 3

Dependent variable was fibrinogen, with independent variables hs-cTnT, NT-proBNP, hsCRP, HbA_1c_, Cholesterol/HDL ratio, VAT and a priori covariates.

#### Model 4

Dependent variable was hsCRP, with independent variables hs-cTnT, NT-proBNP, fibrinogen, HbA_1c_, Cholesterol/HDL ratio, VAT and a priori covariates.

Statistical significance was set at *P* ≤ 0.05 (two-tailed). All regression models were verified for the absence of collinearity. Analyses were performed with STATA16 (College Station, Texas).

### Ethics approval and consent to participate

The study protocol was approved by the Institutional Review Board of the National Institutes of Health, National Institute of Diabetes, Digestive and Kidney Diseases (NIDDK) (ClinicalTrials.gov Identifier: NCT00001853). All participants completed and signed informed consent prior to enrollment in the study. All methods were performed according to the relevant ethical, clinical and scientific research guidelines and regulations.

## Results

Of the 450 African-born Blacks, 65% were male, mean age 39 ± 10 years (range 20–65 years), mean BMI 28 ± 5 kg/m^2^ (range 18–42 kg/m^2^). African regions of origin were: West Africa 49% (222/450), Central Africa 18% (80/450) and East Africa 33% (148/450). The 6 participants from Southern African countries were grouped with the Central Africa region (Supplementary Table 1). Most of our participants were male (65%). The prevalence of Abnl-GT did not differ by African region of origin. However, Hs-cTnT and NT-proBNP did differ by African region, with East Africa having higher hs-cTnT levels vs West and Central Africa (both *P* < 0.01), and Central Africa having higher NT-proBNP levels vs West and East Africa (both *P* < 0.01). Therefore, African region of origin was added as a covariate in regression model 1 (hs-cTnT) and model 2 (NT-proBNP), for both Abnl-GT groups. The effect of antihypertensive treatment was also assessed. Sensitivity analyses revealed no significant effect of antihypertensive therapy on the overall regression analyses.

### Prevalence, characteristics and etiology of Abnl-GT

Of the 450 participants, 62% were NGT (280/450), and Abnl-GT occurred in 38% (170/450). In the Abnl-GT group, Abnl-GT-β-cell failure occurred in 58% (98/170) and Abnl-GT-IR in 42% (72/170) (Fig. 1). The Abnl-GT-β-cell failure group had significantly lower BMI, WC, VAT and obesity prevalence compared to the Abnl-GT-IR group (all *P* < 0.05). In Abnl-GT-β-cell failure group, fasting glucose, fasting insulin, HbA_1c_, triglycerides, HDL and cholesterol/HDL were also significantly lower compared to Abnl-GT-IR group (all *P* < 0.05) (Table 1). Yet the prevalence of hypertension did not differ significantly between Abnl-GT etiology groups (*P* = 0.260).

**Table 1:** Characteristics for the total cohort and by glucose tolerance status.

Variable^1^	Total cohort N = 450 100%	NGT n = 280 62% (280/450)	Abnl-GT-β-cell failure n = 98 22% (98/450)	Abnl-GT-IR n = 72 16% (72/450)	*P*-value^2^
Age (years)	39 ± 10	37 ± 10	43 ± 10	43 ± 9	0.871
Sex (% male)	65%	64%	67%	67%	0.971
BMI (kg/m^2^)	28 ± 5	27 ± 5	27 ± 4	31 ± 4	< 0.001
Obesity (%)	27%	24%	16%	53%	< 0.001
Waist circumference (cm)	91 ± 12	89 ± 11	91 ± 10	101 ± 10	< 0.001
Visceral adipose tissue (cm^2^)	100 ± 69	81 ± 56	105 ± 64	164 ± 74	< 0.001
Diabetes (%)	7%	0%	14%	26%	0.055
Family history of diabetes (%)	29%	28%	36%	25%	0.122
Systolic BP (mmHg)	119 ± 14	118 ± 13	120 ± 14	123 ± 15	0.245
Diastolic BP (mmHg)	72 ± 9	71 ± 9	73 ± 10	74 ± 9	0.362
Hypertension (%)	13%	10%	16%	23%	0.260
Hypertensive treatment (%)	7%	4%	8%	14%	0.087
eGFR (mL/min.1.73m^2^)^3^	114 ± 18	115 ± 18	113 ± 19	112 ± 20	0.554
Smoking (%)	5%	6%	5%	1%	0.217
**Cardiovascular risk and inflammatory markers**					
hs-cTnT (ng/L)	32.6 (0.5–1209.0)	35.8 (1.1–1208.4)	32.2 (0.5–870.2)	27.2 (0.9–916.8)	0.099
NT-proBNP (pg/mL)	22.1 (0.2–1187.0)	21.4 (0.2–514.3)	22.0 (5.4–1181.8)	25.2 (2.1–1072.2)	0.986
Fibrinogen (pg/mL)	276 (3.5–555.5)	266 (3.5–485.5)	279 (178–381)	291 (194–283)	0.178
hsCRP (mg/L)	0.9 (0.1–36.6)	0.8 (0.1–19.2)	1.0 (0.1–18.7)	2.2 (2.5, 4.9)	0.071
**Glucometabolic profile**					
Fasting glucose (mg/dL)	92 ± 14	87 ± 6	96 ± 10	106 ± 25	< 0.001
Glucose at 2 h (mg/dL)^4^	132 ± 40	110 ± 17	163 ± 37	182 ± 57	< 0.001
Fasting insulin (IU/L)	8 ± 7	7 ± 8	6 ± 2	14 ± 6	< 0.001
Matsuda index	5.22 ± 3.50	6.11 ± 3.77	4.93 ± 2.07	1.98 ± 0.49	< 0.001
Insulin secretion index	0.53 ± 0.30	0.45 ± 0.20	0.66 ± 0.31	0.34 ± 0.17	< 0.001
Oral disposition index	2.77 ± 1.01	2.75 ± 0.96	1.68 ± 0.55	1.31 ± 0.53	< 0.001
**Lipid profile**					
Cholesterol (mg/dL)	167 ± 34	162 ± 33	172 ± 36	177 ± 35	0.464
Triglycerides (mg/dL)	74 ± 37	65 ± 28	75 ± 36	107 ± 52	< 0.001
HDL (mg/dL)	52 ± 14	54 ± 14	53 ± 13	45 ± 12	< 0.001
LDL (mg/dL)	100 ± 31	95 ± 29	106 ± 31	111 ± 35	0.274
Cholesterol/HDL ratio	3.4 ± 1.0	3.2 ± 0.9	3.4 ± 0.9	4.2 ± 1.2	< 0.001

^1^Data expressed as mean ± SD, except for Cardiovascular Risk and Inflammatory Markers which were expressed as the median and interquartile ranges.

^2^Comparisons are of the Abnl-GT-β-cell failure vs Abnl-GT-IR groups; continuous variables compared by unpaired t-tests; categorical variables compared by Chi-square.

^3^eGFR was calculated according to the eGFR-CKD-Epi formula.

^4^Glucose at 2 h post-OGTT.

### Cardiovascular risk and inflammatory markers in NGT and Abnl-GT groups

The median hs-cTnT level for the whole cohort was elevated at 33 ng/L, where the cut-off for subclinical myocardial damage was defined as 14 ng/L. NT-proBNP was also low in the whole cohort (< 50 pg/mL). Within Abnl-GT groups, hs-cTnT trended higher in the Abnl-GT-β-cell failure vs the Abnl-GT-IR group (*P* = 0.099). When comparing the cardiovascular and inflammatory markers, in Abnl-GT-β-cell failure, hsCRP trended lower (*P* = 0.071) versus the Abnl-GT-IR group.

Within the Abnl-GT-β-cell failure group significant correlations (*P* > 0.05) were observed between: hs-cTnT, fibrinogen, hsCRP, HbA_1c_ and the Matsuda Index. NT-proBNP only inversely correlated with the Matsuda Index and oral DI. Fibrinogen correlated with age, sex and HbA_1c_. hsCRP correlated with hs-cTnT, fibrinogen, VAT, HbA_1c_ and Cholesterol/HDL ratio (Supplementary Table 2B).

In the Abnl-GT-IR group, significant correlations (*P* > 0.05) were observed between: hs-cTnT and hsCRP, Cholesterol/HDL ratio and eGFR. NT-proBNP inversely correlated with VAT, HbA_1c_, triglyceride/HDL ratio and linearly with eGFR. Fibrinogen correlated with sex, hypertension status, Cholesterol/HDL ratio and VAT. hsCRP correlated with sex, VAT, HbA_1c_ and inversely correlated with eGFR (Supplementary Table 2C).

### Associations between cardiovascular risk and inflammatory markers, glycemia, lipids and visceral adiposity

#### Table 2A: Abnl-GT-β-cell failure

**Table 2:** Associations between cardiovascular biomarkers, inflammatory markers, HbA1C, cholesterol/HDL ratio and VAT in each Abnl-GT Group by multiple regression.

A. Abnl-GT-β-cell failure (n = 98)					B. Abnl-GT-IR (n = 72)			
Variables^1^	Model 1: hs-cTnT^2, 3, 4^	Model 2: NT-proBNP^2, 3, 4^	Model 3: Fibrinogen^2,3^	Model 4: hsCRP^2,3^	Model 1: hs-cTnT^2, 3, 4^	Model 2: NT-proBNP^2, 3, 4^	Model 3: Fibrinogen^2,3^	Model 4: hsCRP^2,3^
	Adj R^2^ = 42%	Adj R^2^ = 15%	Adj R^2^ = 62%	Adj R^2^ = 55%	Adj R^2^ = 11%	Adj R^2^ = 47%	Adj R^2^ = 41%	Adj R^2^ = 43%
hs-cTnT(ng/L)^3^	–	− 0.19 (− 0.52, − 0.12) *P* = 0.023	4.24 (2.26, 6.03) *P* = 0.006	0.30 (0.19, 1.32) *P* = 0.031	–	− 0.16 (− 0.23, 0.04) *P* = 0.412	0.02 (− 0.01, 0.05) *P* = 0.246	0.02 (− 0.18, 0.15) *P* = 0.843
NT-proBNP (pg/mL)^3^	− 0.19 (− 0.52, − 0.12) *P* = 0.023	–	− 0.92 (− 1.35, 0.05) *P* = 0.665	− 0.09 (− 0.32, 0.14) *P* = 0.455	− 0.16 (− 0.23, 0.04) *P* = 0.412	–	− 2.11 (− 4.45, 0.05) *P* = 0.021	− 0.25 (− 0.55, − 0.09) *P* = 0.045
Fibrinogen (pg/mL)^3^	2.53 (0.65, 4.95) *P* = 0.006	− 0.28 (− 0.43, − 0.06) *P* = 0.655	–	3.73 (2.75, 4.71) *P* < 0.001	1.26 (− 0.90, 3.42) *P* = 0.246	− 3.60 (− 5.43, − 1.50) *P* = 0.009	–	2.83 (1.63, 4.04) *P* < 0.001
hsCRP (ng/L)^3^	0.82 (0.37, 1.26) *P* = 0.013	− 0.08 (− 0.30, 0.14) *P* = 0.445	3.73 (2.75, 4.71) *P* < 0.001	–	0.04 (− 0.44, 0.36) *P* = 0.843	− 0.26 (− 0.52, − 0.05) *P* = 0.045	0.09 (0.05, 0.14) *P* < 0.001	–
HbA1C (%)	0.53 (0.02, 1.04) *P* = 0.041	0.03 (− 0.34, 0.39) *P* = 0.885	1.05 (0.18, 2.11) *P* = 0.011	0.71 (0.37, 1.38) *P* = 0.012	0.09 (− 0.21, 0.40) *P* = 0.543	− 0.22 (− 0.42, − 0.02) *P* = 0.030	− 0.02 (− 0.06, 0.13) *P* = 0.209	0.17 (− 0.3, 0.36) *P* = 0.089
Cholesterol/HDL Ratio	− 0.08 (− 0.38, 0.22) *P* = 0.584	0.06 (− 0.15, 0.26) *P* = 0.592	0.02 (− 0.02, 0.06) *P* = 0.348	0.06 (− 0.16, 0.27) *P* = 0.583	0.92 (0.21, 1.40) *P* = 0.048	− 0.31 (− 1.35, − 0.04) *P* = 0.007	2.53 (0.10, 3.61) *P* = 0.015	4.41 (1.15, 5.24) *P* = 0.005
VAT (cm^2^)	− 0.001 (− 1.05, 0.04) *P* = 0.909	− 0.007 (− 0.001, 0.03) *P* = 0.400	− 0.01 (− 0.25, 0.47) *P* = 0.400	0.07 (0.003, 0.01) *P* = 0.098	0.002 (− 0.004, 0.007) *P* = 0.577	− 0.10 (− 0.30, 0.11) *P* = 0.034	0.01 (− 0.007, 0.06) *P* = 0.084	0.34 (0.01, 0.69) *P* = 0.007

^1^Data provided is β-coefficient and 95% CI.

^2^Natural logarithmic transformed values of hs-cTnT, NT-proBNP, fibrinogen and hsCRP were used.

^3^Models adjusted a priori for: age, sex, hypertension status and smoking.

^4^Model adjusted for African region of origin and eGFR.

*Model 1* (Adj R^2^ = 42%): hs-cTnT associated with fibrinogen (*P* = 0.006), hsCRP (*P* = 0.013), HbA_1c_ (*P* = 0.041) and inversely associated with NT-proBNP (*P* = 0.023).

*Model 2* (Adj R^2^ = 15%): NT-proBNP inversely associated with hs-cTnT (*P* = 0.023).

*Model 3* (Adj R^2^ = 62%): Fibrinogen significantly associated with hs-cTnT (*P* = 0.006), hsCRP (*P* < 0.001) and HbA_1c_ (*P* = 0.011).

*Model 4* (Adj R^2^ = 55%), the associations between hsCRP and hs-cTnT and fibrinogen remained, and additionally, hsCRP associated with HbA_1c_ (*P* = 0.012).

#### Table 2B: Abnl-GT-IR

*Model 1* (Adj R^2^ = 11%): hs-cTnT only associated with cholesterol/HDL (*P* = 0.048).

*Model 2* (Adj R^2^ = 47%): NT-proBNP inversely associated with fibrinogen (*P* = 0.009), hsCRP (*P* = 0.045), HbA_1c_ (*P* = 0.030), cholesterol/HDL (*P* = 0.007) and VAT (*P* = 0.034).

*Model 3* (Adj R^2^ = 41%): Fibrinogen associated with hsCRP (*P* = 0.001), cholesterol/HDL (*P* = 0.015), VAT (borderline at *P* = 0.084) and inversely with NT-proBNP (*P* = 0.021).

*Model 4* (Adj R^2^ = 43%): hsCRP associated with fibrinogen (*P* < 0.001), cholesterol/HDL ratio (*P* = 0.005) and VAT (*P* = 0.007) and inversely associated with NT-proBNP (*P* = 0.045).

In both Abnl-GT-etiologies, additional models were calculated where we adjusted for oral DI and Matsuda Index, yet the manner and significance of these associations remained the same.

## Discussion

The current study indicated different subclinical CVD risk profiles based on Abnl-GT etiology. However, this is a cross-sectional study. Therefore, we can only speculate on possible subclinical CVD mechanisms within these metabolically distinct Abnl-GT phenotypes. By only assessing hyperlipidemia and adiposity, Abnl-GT-β-cell-failure show a less adverse CVD profile than Abnl-GT-IR. However, by expanding the investigation beyond standard markers, using hs-cTnT, NT-proBNP, fibrinogen and hsCRP, an increased, albeit subclinical, CVD risk in Abnl-GT-β-cell-failure became evident. Although the absolute values of these four markers did not differ by Abnl-GT etiology group, the nature and degree of association differed significantly. This is important, as these associations revealed that in Abnl-GT-β-cell-failure CVD mechanisms might mainly involve subclinical myocardial strain and damage, increased coagulability and inflammation—relationships not associated with Cholesterol/HDL or VAT. Additionally, this may indicate that traditional CVD risk estimation, using only lipids and adiposity, may not identify CVD risk in Abnl-GT-β-cell-failure. This highlights the importance of considering Abnl-GT etiology when assessing CVD risk and treatment.

### Abnl-GT-β-cell failure, subclinical myocardial damage, increased coagulability and inflammation

This is the first study to show, a significant, cross-sectional relationship between hs-cTnT, fibrinogen, hsCRP and HbA_1c_, in Abnl-GT-β-cell failure alone.

The degree of hyperglycemia and dyslipidemia was not as severe in Abnl-GT-β-cell failure compared to Abnl-GT-IR^3^. However, the associations of hs-cTnT, fibrinogen and hsCRP with HbA_1c_ were only significant in the Abnl-GT-β-cell failure group. It may suggest that a decline in β-cell function itself may be indicative of subclinical CVD, as a decline in β-cell function has been associated with worse cardiovascular outcomes^33^.

Worse cardiovascular outcomes include subclinical myocardial damage, which has been shown to predict incident diabetes, even in the absence of clinically diagnosed CVD or significant myocardial ischemic scarring^6^. Hs-cTnT is frequently applied as a marker of increased myocardial strain and subclinical myocardial damage^7,8^ and is shown to be an important subclinical CVD biomarker in Black African populations^8,10,14^. In the Africans in America cohort, it is notable that the hs-cTnT for the entire cohort was elevated (median of 33 ng/mL), indicative of subclinical myocardial damage^9^. Elevated hs-cTnT predicted incident diabetes in the Atherosclerosis Risk in Communities Study (ARIC) study^6^, linking hs-cTnT to diabetes. Yet, higher levels of hs-cTnT may reflect a variety of hyperglycemia-related cardiovascular insults, from central myocardial ischemia^6,34^ to peripheral microvascular damage^35^. This connection may be what is observed in our Abnl-GT-β-cell failure group, as the subclinical myocardial damage reflected by hs-cTnT may be partially due to hyperglycemia (glucotoxicity), evidenced by the independent association of hs-cTnT with HbA_1c_. This association was not influenced by, nor seen with Cholesterol/HDL or visceral adiposity. In additional analyses, after correcting for eGFR, this association remained significant.

The independent associations of hs-cTnT with HbA_1c_ support findings from Myhre et al.^7^ and Simic et al.^36^ where higher hs-cTnT associated with higher HbA_1c_.

During sustained hyperglycemia, the fibrinogenic actions of cardiomyocytes are activated^37^. The significant association of fibrinogen with HbA1c and hs-cTnT in the Abnl-GT-β-cell failure group, may reflect an increased cardiac fibrosis risk. In this group, hsCRP also directly associated with HbA_1c_, supporting longitudinal data^38^. Importantly, these hs-cTnT- HbA_1c_, fibrinogen- HbA_1c_ and hsCRP- HbA_1c_ associations were not influenced by Cholesterol/HDL or VAT. This may reflect glucotoxicity-induced subclinical myocardial damage, as well as an increased inflammatory state perpetuated by sustained high glucose levels^39^.

Increased myocardial strain and subclinical myocardial damage, due to sustained hyperglycemia, may invoke increased oxidative stress, disrupted neurohormonal activation and the recruitment of inflammatory cytokines^40^. Importantly, elevated hs-cTnT levels can also be noted in the absence of myocardial damage, due to leakage from the cardiomyocytes upon excessive strain^8^. Aside from such hs-cTnT release, oxidative stress, neurohormonal disruption and increased inflammation may induce hs-cTnT release through increased necrosis, apoptosis and troponin degradation^41^, again, even in the absence of prominent ischemic scarring. Indeed, such an association has been reported to explain the correlation between cardiac troponin release and myocardial fibrosis^40^. In our Abnl-GT-β-cell failure group, this might rationalize the association between hs-cTnT and fibrinogen. However, this hypothesis does not imply that insulin resistance protects against subclinical myocardial damage, but rather emphasizes that other, insulin resistance-independent mechanisms are involved in perpetuating the subclinical myocardial damage observed in this Abnl-β-cell failure group.

Albeit cross-sectional, the significant associations between hs-cTnT, fibrinogen and hsCRP and all three with HbA1c, may hint at the likelihood of shared or overlapping pathological pathways between developing CVD and Abnl-GT within the context of β-cell failure. The association of hsCRP with fibrinogen and hs-cTnT, may indicate that in Abnl-GT-β-cell failure, other triggers are involved in the low-grade inflammatory state associated with subclinical myocardial damage^40^. This shows that in Abnl-GT, inflammation is not solely due to insulin resistance-linked visceral adiposity^41^.

**Figure 2:**
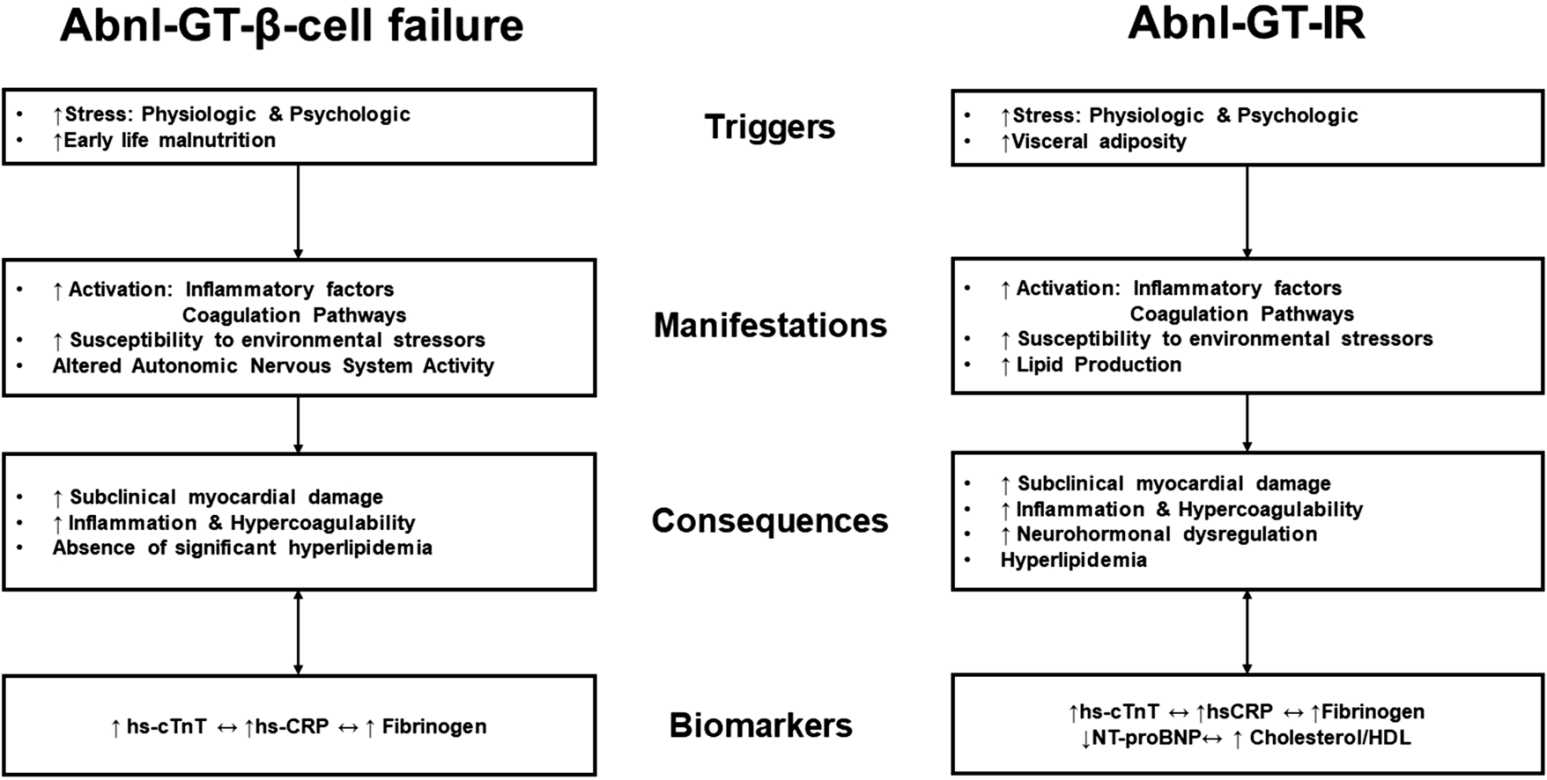
Comparison of pathways, triggers, and consequences which could link CVD risk biomarker profile to etiology of abnormal glucose tolerance.

We speculate, that the relationship between subclinical myocardial damage (hs-cTnT), increased coagulability (fibrinogen) and inflammation (hsCRP) may indicate cumulative subclinical CVD risk, associated with hyperglycemia (HbA1c) in Abnl-GT-β-cell failure. Subclinical cardiac structural changes occur across the continuum of hyperglycemia^7^, and in Abnl-GT-β-cell failure, such changes may be unrelated to Cholesterol/HDL and visceral adiposity. With sustained Abnl-GT due to β-cell failure, CVD risk may increase further (Fig. 2).

### Abnl-GT-IR, adiposity-linked dyslipidemia, inflammation and neurohormonal dysregulation

Contrary to Abnl-GT-β-cell-failure, the CVD risk profile in Abnl-GT-IR is greatly influenced by hyperlipidemia (Cholesterol/HDL ratio) and visceral adiposity (VAT). This is supported by the inverse correlations between a greater degree of IR (lower Matsuda Index) and higher VAT and Cholesterol/HDL. Indeed VAT is the adipose depot most associated with insulin resistance^42^. Increased VAT is an independent predictor of atherogenic dyslipidemia^43^, reflecting that in insulin resistance, altered hepatic lipid production and metabolism may result in dyslipidemia^2,44^.

Both hs-cTnT and fibrinogen associated with cholesterol/HDL and VAT, but not with each other, nor with HbA_1c_—opposite to what was found in the Abnl-GT-β-cell failure group. The relationship between hs-cTnT and hyperlipidemia is known^45,46^ and increased adiposity has been associated with increased inflammation and myocardial damage^47^. Thus, in this cross-sectional study, increased Cholesterol/HDL and VAT were the main factors contributing to the associations between increased myocardial strain, subclinical myocardial damage (hs-cTnT) and increased inflammation (hsCRP) in Abnl-GT-IR.

Additionally, the significant association of hsCRP with hs-cTnT, fibrinogen and VAT, may reflect the chronic low-grade inflammatory state observed with increased adiposity and hyperlipidemia^48^. hsCRP is known to increase in hyperglycemia and is frequently used as a CVD and diabetes risk predictor^7,20^. The associations of hsCRP with hs-cTnT and fibrinogen may also support the involvement of inflammation in subclinical myocardial damage and CVD in the presence of insulin resistance and increased VAT^47,49^. Indeed, even after adjustment for BMI, body fat mass and, alternatively, waist-to-hip ratio, the condition of insulin resistance, rather than hyperglycemia, may predict increased fibrinogen levels in this group^49^. Therefore, the hsCRP-hs-cTnT and hsCRP-fibrinogen associations in Abnl-GT-IR, may mainly be influenced by the condition of IR itself. This possibly indicates that obesity and dyslipidemia may require the state of insulin resistance to trigger or perpetuate the increased inflammation observed in Abnl-GT-IR. Further support for this concept is provided by the inverse correlations between hsCRP and the Matsuda Index and oral DI in Abnl-GT-IR (Supplementary Table 2A)—supporting the association between the degree of insulin resistance and increased inflammation.

Importantly, the NT-proBNP model was highly significant in the Abnl-GT-IR group. Impaired neurohormonal homeostasis, (lower NT-proBNP), is evident in insulin resistance^7^. We found an inverse relationship between HbA_1c_ and NT-proBNP, similar to results reported by Myhre et al.^7^ and Khan et al.^50^. The inverse association between NT-proBNP and hyperglycemia may be influenced by VAT, as increased adiposity can reduce NT-proBNP bio-availability by both reducing its release and increasing its clearance^12^.

NT-proBNP mediates several important cardiovascular effects, including natriuresis, lipid mobilization, vasodilation, and counteracting the renin–angiotensin–aldosterone system and SNS^51^. The reason for this inverse relationship between NT-proBNP and HbA_1c_ reported here and in other population-based studies, is unclear but may directly involve insulin resistance, adipose tissue cytokine overexpression and obesity-linked neurohormonal dysregulation^7,50^. The notion of disrupted neurohormonal homeostasis being linked to worse CVD outcomes, is also supported by the inverse relationship of NT-proBNP with Cholesterol/HDL, fibrinogen and VAT in Abnl-GT-IR.

#### Possible mechanisms of CVD risk in Abnl-GT-IR

Although the current study is cross-sectional, hypothetical mechanisms of subclinical CVD risk in insulin resistance should be mentioned. Dyslipidemia-linked adiposity and the condition of insulin resistance are main factors that contribute to the cardiovascular biomarker associations in Abnl-GT-IR. Indeed, the possible mechanisms of CVD in Abnl-GT-IR may relate to insulin resistance-induced hyperinsulinemia-linked sodium retention, and increased sympathetic activation (low NT-proBNP, associating with lower eGFR), oxidative stress leading to cellular injury and apoptosis (association with hs-cTnT), which may result in decreased cardiac contractility, production of advanced glycation end-products contributing to cardiac fibrosis and inflammation (high fibrinogen and hsCRP)^7,52^. The pathological pathways involved in hyperglycemia and CVD are complex, but Abnl-GT etiology greatly influences the factors and pathways involved in CVD. The hypothetical mechanisms of subclinical CVD in each Abnl-GT etiology are summarized in Fig. 2.

### Strengths and limitations

The strengths of our study included that glucose and insulin levels were obtained during the OGTT. This allowed the calculation of the Matsuda Index to estimate insulin resistance. Additionally, hs-cTnT and hsCRP were analyzed by high-sensitivity methods and all biomarkers showed excellent reproducibility.

Despite the relatively small sample size per Abnl-GT etiology, each group was well-defined, and the regression models carefully constructed. Sensitivity analyses were done in each Abnl-GT etiology group, and correlations performed to consider as many confounding variables as statistically allowed, to determine the independent associations between the cardiovascular biomarkers, inflammatory biomarkers and covariates.

Weaknesses include the cross-sectional design. However, our cohort appears to be representative of African-born Blacks living in the United States. Consistent with known immigration patterns, most of the participants were men from West and Eastern African countries, and BMI was highest in West Africans^22^. Additionally, in our cohort, the prevalence of diabetes was 7% and this is similar to the 8% prevalence of diabetes in African-born Blacks living in Canada, as reported in the Ontario Diabetes Database^53^.

Due to the cross-sectional design, we can only speculate about the possible CVD mechanisms based on Abnl-GT etiology and acknowledge that various factors may specifically influences hs-cTnT and NT-proBNP levels. However, we aimed to statistically account for such influences by the selection of our a priori covariates in the multiple regression models (age, sex, hypertension status, smoking status, African region of origin and eGFR). Additionally, studies should investigate the role of coagulation further by including D-dimer and von Willebrand factor analyses with fibrinogen, particularly in the Abnl-GT-β-cell failure group. Further we can only hypothesize about SNS activity and suggest that assessing heart-rate variability and catecholamines, particularly in Abnl-GT-β-cell failure, might provide more conclusive evidence for this inference.

Nonetheless, the cross-sectional observations made in the study provide the justification for the design of population-based, prospective studies which focus on the different determinants of CVD based on the etiology of Abnl-GT.

## Conclusion

The subclinical CVD risk profile differed based on Abnl-GT etiology. Assessing CVD risk only by adiposity and lipids, Abnl-GT-β-cell-failure appears to have a less adverse CVD profile than Abnl-GT-IR. However, by using hs-cTnT, NT-proBNP, fibrinogen and hsCRP, an increased subclinical CVD risk in Abnl-GT-β-cell-failure became evident. In Abnl-GT-β-cell-failure subclinical CVD mechanisms may involve increased myocardial strain, subclinical myocardial damage, increased coagulability and inflammation—observations not necessarily linked to cholesterol/HDL or VAT, contrary to what was found in Abnl-GT-IR. Thus, traditional CVD risk estimation, using lipids and adiposity alone, may not identify subclinical CVD risk in Abnl-GT-β-cell-failure. The clinical course of CVD may differ based on the etiology of Abnl-GT, indicating the need for modified cardiometabolic treatment regimens ensuring optimized care.

## Supplementary Information

Supplementary Tables.

## Data Availability

Data is available upon reasonable request to Dr Anne E Sumner, MD.

## References

[1] Gerstein HC (2009). Dysglycemia and cardiovascular risk in the general population. Circulation.

[2] Ormazabal V, Nair S, Elfeky O, Aguayo C, Salomon C, Zuniga FA (2018). Association between insulin resistance and the development of cardiovascular disease. Cardiovasc. Diabetol..

[3] Ishimwe MCS, Shoup EM, Osei-Tutu NH, Hormenu T, Patterson AC, Bagheri MH, DuBose CW, Mabundo LS, Ha J, Sherman A, Sumner AE (2021). Beta-cell failure rather than insulin resistance is the major cause of abnormal glucose tolerance in Africans: Insight from the Africans in America study. BMJ Open Diabetes Res Care.

[4] Kibirige D, Lumu W, Jones AG, Smeeth L, Hattersley AT, Nyirenda MJ (2019). Understanding the manifestation of diabetes in sub Saharan Africa to inform therapeutic approaches and preventive strategies: A narrative review. Clin. Diabetes Endocrinol..

[5] Seliger SL, Christernson RH, Kronmal R, Daniels LB, Lima JAC, de Lemos JA, Bertoni A, deFilippi CR (2017). High-sensitivity cardiac troponin T as an early biochemical signature for clinical and subclinical heart failure. Circulation.

[6] Whelton SP, McEvoy JW, Lazo M, Coresh J, Ballantyne CM, Selvin E (2017). High-sensitivity cardiac troponin T (hs-cTnT) as a predictor of incident diabetes in the atherosclerosis risk in communities study. Diabetes Care.

[7] Myhre PL, Lyngbakken MN, Berge T, Roysland R, Aagaard EN, Pervez O (2021). Diagnostic thresholds for pre-diabetes mellitus and diabetes mellitus and subclinical cardiac disease in the general population: Data from the ACE 1950 study. J. Am. Heart Assoc..

[8] Wentzel A, Malan L, von Kanel R, Malan NT (2019). Ethnicity-specific changes in cardiac troponin T in response to acute mental stress and ethnicity-specific cutpoints for the R wave of the aVL lead. Am. J. Epidemiol..

[9] Wang X, Wang P, Cao R, Yang X, Xiao W, Zhang Y (2021). High-sensitivity cardiac troponin T is a risk factor for major adverse cardiovascular events and all-cause mortality: A 9.5-year follow-up study. Cardiol. Res. Pract..

[10] van Vuren EJ, von Kaenel R, Cockeran M, Malan NT (2016). Hyperpulsatile pressure, systemic inflammation and cardiac stress are associated with cardiac wall remodeling in an African male cohort: The SABPA study. Hypertens. Res..

[11] Nicoli CDPTB, Long DL, Judd SE, McClure LA, Arora P, Cushman M (2021). N-termianl Pro-B-type natriuretic peptide and longitudinal risk of hypertension. Am. J. Hypertens..

[12] Jordan JBAL, Melander O, Moro C (2018). Natriuretic peptides in cardiovascular and metabolic crosstalk. Hypertension.

[13] Liu HH, Cao YX, Jin JL, Guo YL, Zhu CG, Wu NQ (2021). Prognostic value of NT-proBNP in patients with chronic coronary syndrome and normal left ventricular systolic function according to glucose status: A prospective cohort study. Cardiovasc. Diabetol..

[14] van Vuren EJ, Malan L, von Kanel R, Magnusson M, Lammertyn L, Malan NT (2019). Prospective associations between cardiac stress, glucose dysregulation and executive cognitive function in Black men: The sympathetic activity and ambulatory blood pressure in Africans study. Diab. Vasc. Dis. Res..

[15] Blankenberg S, McQueen MJ, Smieja M, Pogue J, Balion C, Lonn E, Yusuf S (2006). Comparative impact of multiple biomarkers and N-terminal pro-brain natriuretic peptide in the context of conventional risk factors for the prediction of recurrent cardiovascular events in the heart outcomes prevention evaluation (HOPE) study. Circulation.

[16] Lasse M, Pilbrow AP, Kleffmann T, Andersson Overstrom E, von Zychlinski A, Frampton CMA (2021). Fibrinogen and hemoglobin predict near future cardiovascular events in asymptomatic individuals. Sci. Rep..

[17] Davalos D, Akassoglou K (2012). Fibrinogen as a key regulator of inflammation in disease. Semin. Immunopathol..

[18] Zhang L, Xu C, Liu J, Bai X, Li R, Wang L (2019). Baseline plasma fibrinogen is associated with haemoglobin A1c and 2-year major adverse cardiovascular events following percutaneous coronary intervention in patients with acute coronary syndrome: A single-centre, prospective cohort study. Cardiovasc. Diabetol..

[19] Aso Y, Wakabayashi S, Nakano T, Yamamoto R, Takebayashi K, Inukai T (2006). High serum high-sensitivity C-reactive protein concentrations are associated with relative cardiac sympathetic overactivity during the early morning period in type 2 diabetic patients with metabolic syndrome. Metabolism.

[20] Pradhan ADMJE, Rifai N, Buring JE, Ridker PM (2001). C-reactive protein, interleukin 6, and risk of developing type 2 diabetes mellitus. JAMA.

[21] Shoup EM, Hormenu T, Osei-Tutu NH, Ishimwe MCS, Patterson AC, DuBose CW (2020). Africans who arrive in the United States before 20 years of age maintain both cardiometabolic health and cultural identity: Insight from the Africans in America study. Int. J. Environ. Res. Public Health.

[22] Waldman ZC, Schenk BR, Duhuze Karera MG, Patterson AC, Hormenu T, Mabundo LS (2022). Sleep and economic status are linked to daily life stress in African-Born blacks living in America. Int. J. Environ. Res. Public Health.

[23] Hobabagabo AF, Osei-Tutu NH, Hormenu T, Shoup EM, DuBose CW, Mabundo LS (2020). Improved detection of abnormal glucose tolerance in Africans: The value of combining hemoglobin A1c with glycated albumin. Diabetes Care.

[24] Briker SM, Aduwo JY, Mugeni R, Horlyck-Romanovsky MF, DuBose CW, Mabundo LS (2019). A1C Underperforms as a diagnostic test in Africans even in the absence of nutritional deficiencies, anemia and hemoglobinopathies: Insight From the Africans in America Study. Front. Endocrinol. (Lausanne).

[25] Whelton PK, Carey RM, Aronow WS, Casey DE, Collins KJ, Dennison Himmelfarb C (2018). 2017 ACC/AHA/AAPA/ABC/ACPM/AGS/APhA/ASH/ASPC/NMA/PCNA Guideline for the Prevention, Detection, Evaluation, and Management of High Blood Pressure in Adults: A Report of the American College of Cardiology/American Heart Association Task Force on Clinical Practice Guidelines. Hypertension.

[26] Organization WH. *Obesity and Overweight [Fact Sheet]*. WHO. https://www.who.int/news-room/fact-sheets/detail/obesity-and-overweight. (2020) (Accessed 1 April 2020).

[27] Patry-Parisien JSM, Bryan S (2012). Comparison of waist circumference using the World Health Organization and National Institutes of Health protocols. Health Rep..

[28] Levey ASSLA, Schmid CH, Zhang Y, Castro AF, Feldman HI, Kusek JW, Eggers P, Van Lente F, Greene T (2009). A new equation to calculate estimated glomerular filtration rate. Ann. Intern. Med..

[29] O'Connor MY, Thoreson CK, Ricks M, Courville AB, Thomas F, Yao J (2014). Worse cardiometabolic health in African immigrant men than African American men: Reconsideration of the healthy immigrant effect. Metab. Syndr. Relat. Disord..

[30] Matsuda MDRA (1999). INsulin sensitivity indices obtained from the oral glucose tolerance testing: Comparison with the euglycemic insulin clamp. Diabetes Care.

[31] Utzschneider KMPR, Faulenbach MV, Tong J, Carr DB, Boyko EJ (2009). Oral disposition index predicts the development of future diabetes above and beyond fasting and 2-h glucose levels. Diabetes Care.

[32] Friedewald WTLRI, Fredrickson DS (1972). Estimation of the concentration of low-density lipoprotein cholesterol in plasma, without use of the preparative ultracentrifuge. Clin. Chem..

[33] Curtis LHHBG, Bethel MA, Anstrom KJ, Liao L, Gottdiener JS, Schul KA (2008). Pancreatic B-cell function as a predictor of cardiovascular outcomes and costs: Findings from the cardiovascular health study. Curr. Med. Res. Opin..

[34] Ndumele CE, Coresh J, Lazo M, Hoogeveen RC, Blumenthal RS, Folsom AR (2014). Obesity, subclinical myocardial injury, and incident heart failure. JACC Heart Fail..

[35] Wentzel AML, Smith W, von Känel R, Malan NT (2019). Retinal vasculature reactivity during flicker light provocation, cardiac stress and stroke risk in Africans: The SABPA study. Transl. Stroke Res..

[36] Simic S, Svagusa T, Prkacin I, Bulum T (2019). Relationship between hemoglobin A1c and serum troponin in patients with diabetes and cardiovascular events. J. Diabetes Metab. Disord..

[37] Russo I, Frangogiannis NG (2016). Diabetes-associated cardiac fibrosis: Cellular effectors, molecular mechanisms and therapeutic opportunities. J. Mol. Cell Cardiol..

[38] Ahmadi-Abhari S, Kaptoge S, Luben RN, Wareham NJ, Khaw K (2015). Longitudinal association of C-reactive protein and Haemoglobin A1c over 13 years: The European Prospective Investigation into Cancer—Norfolk study. Cardiovasc. Diabetol..

[39] Battault S, Renguet E, Van Steenbergen A, Horman S, Beauloye C, Bertrand L (2020). Myocardial glucotoxicity: Mechanisms and potential therapeutic targets. Arch. Cardiovasc. Dis..

[40] Marzban L (2015). New insights into the mechanisms of islet inflammation in type 2 diabetes. Diabetes.

[41] Sun Q, Li J, Gao F (2014). New insights into insulin: The anti-inflammatory effect and its clinical relevance. World J. Diabetes.

[42] Kabakambira DJ, Briker SM, Courville AB, Mabundo LS, DuBose CW, Chung ST, Eckel RH, Sumner AE (2018). Do current guidelines for waist circumference apply to black Africans? Prediction of insulin resistance by waist circumference among Africans living in America. BMJ Glob. Health.

[43] Hwang YCFWY, Hayashi T, Kahn SE, Leonetti DL, Boyko EJ (2015). Increased visceral adipose tissue is an independent predictor for future development of atherogenic dyslipidemia. J. Clin. Endocrinol. Metab..

[44] Liu L, Feng J, Zhang G, Yuan X, Li F, Yang T (2018). Visceral adipose tissue is more strongly associated with insulin resistance than subcutaneous adipose tissue in Chinese subjects with pre-diabetes. Curr. Med. Res. Opin..

[45] Welsh P, Preiss D, Hayward C, Shah ASV, McAllister D, Briggs A (2019). Cardiac troponin T and troponin I in the general population. Circulation.

[46] White HD (2020). Clinically important improvements in risk assessment by adding high-sensitivity troponin level to cholesterol guidelines. JAMA Cardiol..

[47] Nath D, Shivashekar M, Vinodhini VM (2019). Fibrinogen levels in obese and normal individuals. J. Clin. Diagn. Res..

[48] Galmes S, Cifre M, Palou A, Oliver P, Serra F (2019). A genetic score of predisposition to low-grade inflammation associated with obesity may contribute to discern population at risk for metabolic syndrome. Nutrients.

[49] Raynaud EP-MA, Brun JF, Aissa-Benhaddad A, Fedou C, Mercier J (2000). Relationships between fibrinogen and insulin resistance. Atherosclerosis.

[50] Khan AMCS, Magnusson M, Larson MG, Newton-Cheh C, McCabe EL, Coviello AD, Florez JC, Fox CS, Levy D, Robins SJ, Arora P, Bhasin S, Lam CSP, Vasan RS, Melander O, Wang TJ (2011). Cardiac natriuretic peptides, obesity and insulin resistance: Evidence from two community-based studies. J. Clin. Endocrinol. Metab..

[51] Gupta DK, Wang TJ (2015). Natriuretic peptides and cardiometabolic health. Circ. J..

[52] van Vuren EJ, Malan L, Cockeran M, Scheepers JD, Oosthuizen W, Malan NT (2016). Fibrosis and coronary perfusion—A cardiovascular disease risk in an African male cohort: The SABPA study. Clin. Exp. Hypertens..

[53] Creatore MI, Moineddin R, Booth G, Manuel DH, DesMeules M, McDermott S (2010). Age- and sex-related prevalence of diabetes mellitus among immigrants to Ontario, Canada. CMAJ.

