# Supplementary material for: The Africans in America study demonstrates that subclinical cardiovascular risk differs by etiology of abnormal glucose tolerance
Source: Sci Rep. 2022 Oct 10;12:16947. doi: 10.1038/s41598-022-19917-8 (PMC9551031; doi:10.1038/s41598-022-19917-8)

**Supplementary Tables**

**Supplementary Table 1: Characteristics according to African Region of origin**

| **Variable^1^** | **Total Cohort**  **N=450**  **100%** | **West Africa^2^**  **n=222**  **49% (222/450)** | **Central Africa^2^**  **n=80**  **18% (80/450)** | **East Africa^2^**  **n=148**  **33% (148/450)** |
| --- | --- | --- | --- | --- |
| **Age (years)** | 39±10 | 40±11 | 39±10 | 38±9 |
| **Sex (% Male)** | 65% | 67% | 72% | 59% |
| **BMI (kg/m^2^)** | 28±5 | 28±5 | 28±5 | 27±4 |
| **Obesity (%)** | 27% | 31% | 29% | 22% |
| **WC (cm)** | 91±12 | 92±12 | 90±11 | 91±12 |
| **VAT (cm^2^)** | 100±68 | 95±63 | 107±72 | 103±73 |
| **Diabetes (%)** | 7% | 8% | 8% | 7% |
| **Prediabetes (%)** | 30% | 27% | 38% | 30% |
| **Abnormal Glucose Tolerance (%)** | 37% | 35% | 46% | 37% |
| **Family History of T2D (%)** | 29% | 29% | 28% | 28% |
| **Systolic BP (mmHg)** | 119±14 | 120±14 | 122±12 | 116±14 |
| **Diastolic BP (mmHg)** | 72±10 | 72±10 | 73±10 | 71±9 |
| **Hypertension (%)** | 13% | 15% | 14% | 11% |
| **eGFR (mL/min.1.73m^2^)^3^** | 114±18 | 108±17 | 114±17 | 122±17 |
| **Smoking (%)** | 5% | 7% | 5% | 5% |
| **hs-cTnT (ng/L)** | 32.6 (0.5-1209.0) | 31.8 (0.5-1209.0) | 37.2 (1.5-689.2) | 34.2 (3.0-914.6)b, c^**^ |
| **NT-proBNP (pg/mL)** | 22.1 (0.2-1187.0) | 20.1 (0.2-1074.0)a^**^ | 19.6 (2.7-1184.5) | 25.2 (2.9-360.5)c^**^ |
| **Fibrinogen (pg/mL)** | 276 (3.5-555.5) | 276 (3.5-555.5) | 268.5 (128.0-304.0) | 276 (160, 317) |
| **hsCRP (mg/L)** | 0.9 (0.1-36.6) | 0.8 (1.6, 2.6) | 0.9 (0.4, 14.1) | 1.1 (0.1, 18.3) |
| **Fasting plasma glucose (mg/dL)** | 92±14 | 93±16 | 92±10 | 92±9 |
| **Glucose at 2h (mg/dL)^4^** | 133±43 | 134±48 | 135±32 | 132±40 |
| **Fasting plasma insulin (UI/L)** | 8±7 | 9±9 | 8±6 | 7±4 |
| **Matsuda Index** | 5.22±3.50 | 5.20±3.47 | 5.34±4.24 | 5.17±2.96 |
| **Insulin Secretion Index** | 0.53±0.30 | 0.49±0.28 | 0.42±0.31 | 0.48±0.29 |
| **Oral Disposition Index** | 2.30±1.01 | 2.55±1.08 | 2.24±1.06 | 2.48±1.12 |
| **Cholesterol (mg/dL)** | 167±35 | 169±35 | 164±32 | 165±35 |
| **Triglycerides (mg/dL)** | 74±38 | 74±42 | 68±28 | 76±34 |
| **HDL (mg/dL)** | 52±14 | 53±14 | 52±15 | 51±14 |
| **LDL (mg/dL)** | 100±31 | 101±31 | 99±30 | 100±32 |

^1^Data expressed as either mean±SD or percentages, except for Cardiovascular Risk and Inflammatory Markers which were expressed as the median and interquartile ranges

^2^Comparisons were between the three African regions of origin: continuous variables compared by One-Way ANOVA with Bonferroni corrections for multiple comparisons; a: West vs Central, b: West vs East, c: Central vs East, **P* ≤0.05, ***P* ≤0.01, ****P* ≤0.001, categorical variables compared by: chi-square.

^3^eGFR was calculated according to the eGFR-CKD-Epi formula

^4^Glucose at 2h post-OGTT

**Supplementary Table 2A: Partial correlations between CVD biomarkers and possible *a priori* covariates**

| **Total cohort**  **N=450** | | | | |
| --- | --- | --- | --- | --- |
|  | **hs-cTnT** | **Fibrinogen** | **CRP** | **NT-proBNP** |
| **hs-cTnT** | **1.00** | 0.10  *P*=0.042 | 0.05  *P*=0.229 | 0.09  *P*=0.854 |
| **Fibrinogen** | 0.10  *P*=0.042 | **1.00** | 0.55  *P*<0.001 | 0.02  *P*=0.735 |
| **hsCRP** | 0.05  *P*=0.229 | 0.55  *P*<0.001 | **1.00** | 0.48  *P*=0.313 |
| **NT-proBNP** | 0.09  *P*=0.854 | 0.02  *P*=0.735 | 0.48  *P*=0.313 | **1.00** |
| **Age** | -0.13  *P*=0.007 | 0.22  *P*<0.001 | 0.22  *P*<0.001 | 0.09  *P*=0.050 |
| **Sex** | 0.10  *P*=0.032 | 0.36  *P*<0.001 | 0.16  *P*<0.001 | 0.02  *P*=0.633 |
| **Hypertension Status** | -0.08  *P*=0.098 | -0.10  *P*=0.214 | -0.04  *P*=0.451 | -0.02  *P*=0.624 |
| **VAT** | -0.10  *P*=0.073 | 0.15  *P*=0.002 | 0.24  *P*<0.001 | -0.12  *P*=0.010 |
| **A1C** | -0.05  *P*=0.313 | 0.16  *P*<0.001 | 0.17  *P*<0.001 | 0.03  *P*=0.542 |
| **Total cholesterol/HDL** | 0.01  *P*=0.812 | 0.15  *P*=0.001 | 0.15  *P*=0.001 | 0.01  *P*=0.989 |
| **Triglyceride/HDL** | 0.05  *P*=0.325 | 0.13  *P*=0.009 | 0.13  *P*=0.009 | 0.05  *P*=0.281 |
| **eGFR** | 0.10  *P*=0.044 | 0.03  *P*=0.598 | 0.05  *P*=0.342 | 0.10  *P*=0.035 |
| **Matsuda Index** | -0.15  *P*=0.032 | -0.26  *P*=0.002 | 0.35  *P*=0.032 | -0.28  *P*=0.051 |
| **Oral Disposition Index** | 0.12  *P*=0.078 | 0.22  *P*=0.089 | 0.41  *P*=0.007 | -0.29  *P*=0.001 |
| **Socio-economic and Health Behaviors** | | | | |
| **Physical activity** | 0.06  *P*=0.334 | -0.08  *P*=0.167 | 0.04  *P*=0.523 | -0.05  *P*=0.369 |
| **Income** | 0.05  *P*=0.256 | 0.11  *P*=0.018 | -0.003  *P*=0.942 | -0.12  *P*=0.012 |
| **Education** | 0.08  *P*=0.872 | -0.10  *P*=0.076 | -0.13  *P*=0.008 | -0.04  *P*=0.414 |
| **Drinking** | 0.02  *P*=0.697 | -0.03  *P*=0.490 | 0.08  *P*=0.107 | 0.01  *P*=0.859 |
| **Smoking** | -0.09  *P*=0.053 | 0.04  *P*=0.408 | -0.14  *P*=0.004 | -0.02  *P*=0.699 |

**Supplementary Table 2B: Partial correlations between CVD biomarkers and *a priori* covariates in Abnl-GT-β-cell failure**

| **Abnl-GT-β-cell failure**  **N=98** | | | | |
| --- | --- | --- | --- | --- |
|  | **cTnT** | **Fibrinogen** | **CRP** | **NT-proBNP** |
| **cTnT** | 1.00 | 0.14  *P*=0.024 | 0.10  *P*=0.040 | -0.04  *P*=0.067 |
| **Fibrinogen** | 0.14  *P*=0.024 | 1.00 | 0.59  *P*<0.001 | -0.02  *P*=0.863 |
| **CRP** | 0.10  *P*=0.040 | 0.59  *P*<0.001 | 1.00 | -0.04  P=0.684 |
| **NT-proBNP** | -0.04  *P*=0.067 | -0.02  *P*=0.863 | -0.04  *P*=0.684 | 1.00 |
| **Age** | -0.002  *P*=0.987 | 0.25  *P*=0.018 | 0.09  *P*=0.390 | 0.03  *P*=0.806 |
| **Sex** | 0.07  *P*=0.522 | 0.49  *P*<0.001 | 0.11  *P*=0.314 | -0.05  *P*=0.655 |
| **Hypertension Status** | 0.08  *P*=0.044 | 0.10  *P*=0.332 | 0.14  *P*=0.170 | 0.007  *P*=0.947 |
| **VAT** | 0.004  *P*=0.969 | 0.02  *P*=0.849 | 0.24  *P*=0.025 | 0.01  *P*=0.895 |
| **A1C** | 0.13  *P*=0.021 | 0.26  *P*=0.012 | 0.20  *P*=0.058 | -0.04  *P*=0.680 |
| **Total cholesterol/HDL** | 0.11  *P*=0.307 | 0.16  *P*=0.128 | 0.24  *P*=0.020 | 0.12  *P*=0.264 |
| **Triglyceride/HDL** | 0.12  *P*=0.265 | 0.02  *P*=0.819 | 0.23  *P*=0.029 | 0.08  *P*=0.439 |
| **eGFR** | 0.07  *P*=0.506 | 0.01  *P*=0.958 | 0.001  *P*=0.986 | 0.02  *P*=0.851 |
| **Matsuda Index** | 0.37  *P*=0.002 | 0.42  *P*=0.021 | -0.36  *P*=0.003 | -0.22  *P*=0.061 |
| **Oral Disposition Index** | -0.15  *P*=0.088 | -0.19  *P*=0.080 | -0.39  *P*=0.012 | -0.31  *P*=0.007 |
| **Socio-economic and Health Behaviors** | | | | |
| **Physical activity** | 0.51  *P*<0.001 | -0.23  *P*=0.067 | -0.18  *P*=0.170 | 0.06  *P*=0.629 |
| **Income** | 0.08  *P*=0.445 | 0.09  *P*=0.379 | -0.16  *P*=0.123 | -0.16  *P*=0.117 |
| **Education** | 0.03  *P*=0.756 | -0.03  *P*=0.795 | -0.15  *P*=0.162 | -0.003  *P*=0.972 |
| **Drinking** | 0.03  *P*=0.770 | 0.14  *P*=0.185 | 0.20  *P*=0.053 | -0.09  *P*=0.411 |
| **Smoking** | -0.07  *P*=0.496 | 0.02  *P*=0.053 | 0.11  *P*=0.028 | -0.03  *P*=0.807 |

**Supplementary Table 2C: Partial correlations between CVD biomarkers and *a priori* covariates in Abnl-GT-IR**

| **Abnl-GT-IR**  **N=72** | | | | |
| --- | --- | --- | --- | --- |
|  | **cTnT** | **Fibrinogen** | **CRP** | **NT-proBNP** |
| **cTnT** | 1.00 | 0.19  P=0.104 | 0.20  P=0.009 | -0.02  P=0.084 |
| **Fibrinogen** | 0.19  *P*=0.104 | 1.00 | 0.55  *P*<0.001 | -0.13  *P*=0.054 |
| **CRP** | 0.20  *P*=0.009 | 0.55  *P*<0.001 | 1.00 | 0.07  *P*=0.538 |
| **NT-proBNP** | -0.02  *P*=0.084 | -0.13  *P*=0.054 | 0.07  *P*=0.538 | 1.00 |
| **Age** | -0.14  *P*=0.254 | -0.13  *P*=0.290 | -0.01  *P*=0.942 | 0.16  *P*=0.186 |
| **Sex** | 0.01  *P*=0.934 | 0.40  *P*<0.001 | 0.31  *P*=0.007 | -0.04  *P*=0.742 |
| **Hypertension Status** | 0.08  *P*=0.496 | 0.31  *P*=0.007 | 0.06  *P*=0.658 | 0.09  *P*=0.455 |
| **VAT** | 0.04  *P*=0.712 | 0.16  *P*=0.017 | 0.30  *P*=0.009 | -0.20  *P*=0.009 |
| **A1C** | -0.04  *P*=0.743 | -0.07  *P*=0.586 | 0.11  *P*=0.038 | -0.04  *P*=0.076 |
| **Total cholesterol/HDL** | 0.16  *P*=0.018 | 0.35  *P*=0.036 | 0.01  *P*=0.955 | -0.13  *P*=0.271 |
| **Triglyceride/HDL** | 0.02  *P*=0.898 | -0.14  *P*=0.243 | -0.09  *P*=0.442 | -0.55  *P*=0.006 |
| **eGFR** | 0.26  *P*=0.025 | 0.13  *P*=0.324 | -0.43  *P*<0.001 | 0.39  *P*<0.001 |
| **Matsuda Index** | -0.22  *P*=0.261 | -0.31  *P*=0.187 | -0.34  *P*=0.002 | -0.31  *P*=0.004 |
| **Oral Disposition Index** | -0.09  *P*=0.487 | -0.11  *P*=0.899 | -0.38  *P*<0.001 | -0.41  *P*=0.001 |
| **Socio-economic and Health Behaviors** | | | | |
| **Physical activity** | 0.18  P=0.212 | 0.14  P=0.326 | 0.24  P=0.082 | -0.10  P=0.477 |
| **Income** | 0.05  P=0.648 | 0.10  P=0.388 | 0.01  P=0.947 | -0.22  P=0.061 |
| **Education** | -0.15  P=0.229 | -0.08  P=0.495 | -0.12  P=0.317 | 0.06  P=0.621 |
| **Drinking** | -0.07  P=0.522 | -0.17  P=0.143 | 0.05  P=0.681 | 0.08  P=0.522 |
| **Smoking** | -0.02  P=0.886 | -0.06  P=0.603 | -0.05  P=0.650 | -0.03  P=0.803 |

**Supplementary Table 3: Post-Hoc Power Analysis**


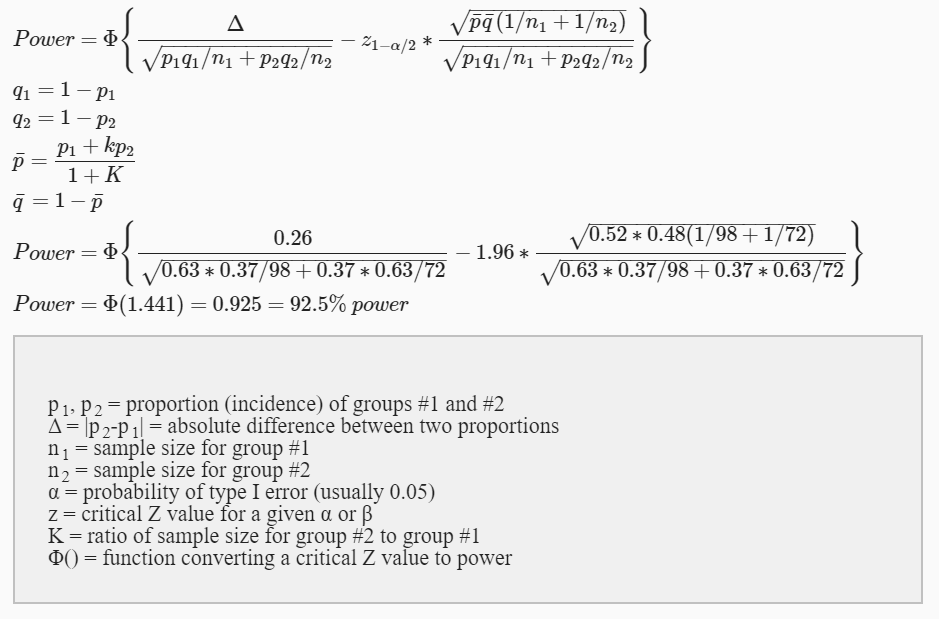

Supplement: Supplementary file 1 — Supplementary Tables. [file 41598_2022_19917_MOESM1_ESM.docx]
